# A single natural RNA modification can destabilize a U•A-T-rich RNA•DNA-DNA triple helix

**DOI:** 10.1261/rna.079244.122

**Published:** 2022-09

**Authors:** Charlotte N. Kunkler, Grace E. Schiefelbein, Nathan J. O'Leary, Phillip J. McCown, Jessica A. Brown

**Affiliations:** 1 Department of Chemistry and Biochemistry, University of Notre Dame, Notre Dame, Indiana 46556, USA; 2 Michigan Medicine, Department of Internal Medicine, Division of Nephrology, University of Michigan, Ann Arbor, Michigan 48109, USA

**Keywords:** long noncoding RNA, RNA modification, triple helix

## Abstract

Recent studies suggest noncoding RNAs interact with genomic DNA, forming RNA•DNA-DNA triple helices, as a mechanism to regulate transcription. One way cells could regulate the formation of these triple helices is through RNA modifications. With over 140 naturally occurring RNA modifications, we hypothesize that some modifications stabilize RNA•DNA-DNA triple helices while others destabilize them. Here, we focus on a pyrimidine-motif triple helix composed of canonical U•A-T and C•G-C base triples. We employed electrophoretic mobility shift assays and microscale thermophoresis to examine how 11 different RNA modifications at a single position in an RNA•DNA-DNA triple helix affect stability: 5-methylcytidine (m^5^C), 5-methyluridine (m^5^U or rT), 3-methyluridine (m^3^U), pseudouridine (Ψ), 4-thiouridine (s^4^U), *N*^6^-methyladenosine (m^6^A), inosine (I), and each nucleobase with 2′-*O*-methylation (Nm). Compared to the unmodified U•A-T base triple, some modifications have no significant change in stability (Um•A-T), some have ∼2.5-fold decreases in stability (m^5^U•A-T, Ψ•A-T, and s^4^U•A-T), and some completely disrupt triple helix formation (m^3^U•A-T). To identify potential biological examples of RNA•DNA-DNA triple helices controlled by an RNA modification, we searched RMVar, a database for RNA modifications mapped at single-nucleotide resolution, for lncRNAs containing an RNA modification within a pyrimidine-rich sequence. Using electrophoretic mobility shift assays, the binding of DNA-DNA to a 22-mer segment of human lncRNA Al157886.1 was destabilized by ∼1.7-fold with the substitution of m^5^C at known m^5^C sites. Therefore, the formation and stability of cellular RNA•DNA-DNA triple helices could be influenced by RNA modifications.

## INTRODUCTION

Pyrimidine-motif RNA and DNA triple helices were shown to form in vitro over 60 yr ago (Felsenfeld and Rich 1957; Felsenfeld et al. 1957; Lipsett 1964; Riley et al. 1966; Morgan and Wells 1968). In such structures, a single strand of pyrimidine-rich RNA or DNA binds in a parallel orientation along the major groove of a purine-rich double-strand (ds) of Watson–Crick base-paired RNA or DNA, forming stacked base triples (Arnott and Bond 1973; Arnott and Selsing 1974; Arnott et al. 1976). Since then, pyrimidine-motif triple helices, defined herein as three or more consecutive base triples, have been structurally validated in RNAs across all domains of life (Brown 2020). However, the largest pool of triple helices in vivo may be RNA•DNA-DNA triple helices (R•D-D, where “•” and “-” represent Hoogsteen and Watson-Crick interactions, respectively), which form between noncoding RNAs (ncRNAs) and genomic DNA (gDNA). Many proposed ncRNA•gDNA triple helices form between long noncoding RNA (lncRNA) and promoter regions of gDNA to regulate gene expression by recruiting chromatin-modifying enzymes (Sentürk Cetin et al. 2019). For example, the lncRNA Fendrr (Fetal lethal noncoding developmental regulatory RNA) forms a pyrimidine-motif triple helix within two different gDNA regions to silence *Foxf1* and *Pitx2* through the recruitment of chromatin-modifying complexes PRC2 and TrxG/MLL, respectively (Grote and Herrmann 2013; Grote et al. 2013). Additionally, the lncRNA Khps1 (antisense to SPHK1) recruits the chromatin-modifying enzymes E2F1 and p300 to express SPHK1 (sphingosine kinase 1) (Postepska-Igielska et al. 2015; Blank-Giwojna et al. 2019). Considering that there are at least 27,000 genes encoding lncRNAs in human gDNA, the R•D-D triple helix could represent a large percentage of the triple helices in human cells and could be a general mechanism for RNA to regulate gene expression (Hon et al. 2017; Soibam 2017; Sentürk Cetin et al. 2019).

We recently determined the relative stability of 16 different base triples at a single position in an R•D-D triple helix (Kunkler et al. 2019). Though the most stable base triples for this triple helix are the canonical U•A-T and C•G-C base triples, other noncanonical base triples also allow for the formation of the triple helix (Kunkler et al. 2019). However, the effect of RNA modifications at a single position within an R•D-D triple helix has not yet been systematically studied. Over 140 different modifications of cellular RNA are known, with at least 12 identified in human lncRNAs (McCown et al. 2020; Yang et al. 2020b). One primary function of RNA modifications is to alter the stability of RNA secondary and tertiary structures (McCown et al. 2020). Therefore, RNA modifications may function as “switches” to regulate the formation of R•D-D triple helices in vivo and, in turn, regulate gene expression. Some modifications have been shown to change the stability of DNA•DNA-DNA (D•D-D) and RNA•RNA-RNA (R•R-R) triple helices in vitro, suggesting a stability change for R•D-D triple helices (Lee et al. 1984; Povsic and Dervan 1989; Xodo et al. 1991; Shimizu et al. 1992; Wang and Kool 1995; Wang et al. 1997; Zhou et al. 2013; Jacob et al. 2017). For example, 5-methylcytidine (m^5^C), one of the most abundant modifications in ncRNAs, was previously shown to stabilize D•D-D triple helices but destabilize R•R-R and R•D-R triple helices (Lee et al. 1984; Povsic and Dervan 1989; Xodo et al. 1991; Wang et al. 1997; Jacob et al. 2017). Another study showed that R•R-R triple helices are destabilized when the entire RNA third strand is 2′-*O*-methylated, while R•D-D triple helices are stabilized by an RNA third strand composed entirely of 2′-*O*-methylated uridines or cytidines (i.e., Um•A-U/T and Cm•G-C, respectively) (Shimizu et al. 1992; Wang and Kool 1995; Zhou et al. 2013). Because R•D-D triple helices have been implicated to play a role in transcription regulation for a variety of genes via ncRNA•gDNA interactions, the effect of RNA modifications on R•D-D triple helix formation is an exciting possibility to explore (Li et al. 2016).

For this study, 11 naturally occurring RNA modifications were chosen due to their commercial availability and, for most, their presence in human lncRNAs (underlined modifications have not been detected): 2′-*O*-methylcytidine (Cm), 5-methylcytidine (m^5^C), 2′-*O*-methyluridine (Um), 5-methyluridine (m^5^U; or ribothymidine, rT), 3-methyluridine (m^3^U), pseudouridine (Ψ), 4-thiouridine (s^4^U), 2′-*O*-methyladenosine (Am), *N*^6^-methyladenosine (m^6^A), inosine (I), and 2′-*O*-methylguanosine (Gm). To date, m^3^U has not been identified in human lncRNAs, though it is naturally occurring in human 28S ribosomal RNA (rRNA) (Taoka et al. 2018). Further, we expect a large destabilization of the R•D-D triple helix containing an m^3^U•X-Y base triple due to the methyl group disrupting Hoogsteen interactions, resulting in a destabilized R•D-D triple helix that serves as a control. s^4^U has not been identified in human RNAs, but it is used in RNA–protein, RNA–RNA, and RNA–DNA cross-linking experiments; therefore, the effect on R•D-D triple helix stability may be relevant within an experimental setting (Favre et al. 1986, 1998; Debreuil et al. 1991; Saintomé et al. 1997). Altogether, each of these modifications has been shown to either increase or decrease the stability of other nucleic acid structures, so it is likely that they also affect R•D-D triple helix stability (Lee et al. 1984; Huff and Topal 1987; Povsic and Dervan 1989; Xodo et al. 1991; Shimizu et al. 1992; Wang and Kool 1995; Mills et al. 1996; Kumar and Davis 1997; Wang et al. 1997; Zhou et al. 2013; Jacob et al. 2017; McCown et al. 2020). For each of the 11 RNA modifications, a single base triple in the R•D-D triple helix was varied and the relative stability of the triple helix was measured at a neutral pH using both electrophoretic mobility shift assays (EMSA) and microscale thermophoresis (MST). In general, our R•D-D triple helix with single-site RNA modifications showed no change, minor destabilization, or complete disruption of the triple helix, suggesting some RNA modifications, such as m^5^C in human lncRNA AL157886.1, could perturb the formation of R•D-D triple helices in vivo as a mechanism to regulate gene expression.

## RESULTS AND DISCUSSION

### Most RNA modifications destabilize an R•D-D triple helix

A physiologically relevant three-strand construct was previously used to determine the relative stability of 16 different unmodified R•D-D base triples at a single position using EMSA, concluding that the most stable base triples are the canonical U•A-T and C•G-C base triples (Kunkler et al. 2019). Herein, using the same parent construct and experimental setup, we tested the relative stability of 11 different RNA modifications (Supplemental Fig. S1) within Z•A-T and Z•G-C base triples (where Z = Cm, m^5^C, Um, m^5^U, m^3^U, Ψ, s^4^U, Am, m^6^A, I, Gm) to directly compare the binding to the previously examined unmodified base triples (Kunkler et al. 2019).

**FIGURE 1.**
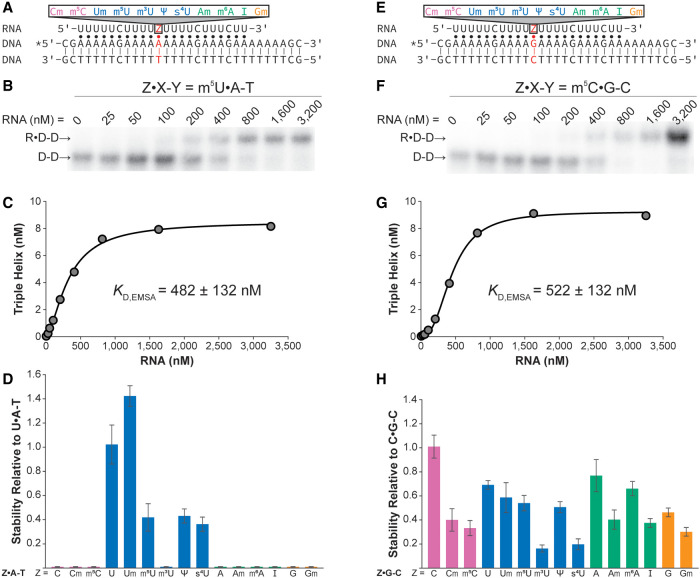
EMSA measurements of the relative stability of 11 modified RNA nucleotides in an R•D-D triple helix. Schematic depicting R•D-D triple helix with the varying position (*A*) Z•A-T and (*E*) Z•G-C in red. The putative Watson–Crick and Hoogsteen interactions are represented by a solid line (|) and a dot (•), respectively. The asterisk (*) denotes the location of the 5'-[^32^P]-radiolabel. Representative gel image for R•D-D triple helix containing the (*B*) m^5^U•A-T base triple and (*F*) m^5^C•G-C base triple, showing a shift from dsDNA (D-D) to triple helix (R•D-D) as increasing amounts of RNA are added. The binding curve generated from the (*C*) m^5^U•A-T and (*G*) m^5^C•G-C gel data points. The relative stability of each R•D-D triple helix with (*D*) Z•A-T base triple and (*H*) Z•G-C base triple is shown as bar plots. The relative stability was calculated as *K*_D, EMSA_(U•A-T)/*K*_D, EMSA_(Z•A-T) in panel *D* and as *K*_D, EMSA_(C•G-C)/*K*_D, EMSA_(Z•G-C) in panel *H*. Each bar color represents a specific RNA nucleobase: pink for C, blue for U, green for A, and orange for G. Reported *K*_D, EMSA_ values and relative stability values are an average of at least three independent replicates, and error bars represent standard deviation.

First, we examined the modified Z•A-T base triples (Fig. 1A). EMSAs were performed, whereby a shift of [^32^P]-radiolabeled dsDNA to a larger R•D-D complex was observed with increasing concentrations of the RNA strand (Fig. 1B). *K*_D, EMSA_ values were determined for each of the 11 modified base triples (Table 1; Fig. 1C, D; Supplemental Table S1), with Um•A-T exhibiting the tightest binding at 132 ± 8 nM and seven modified base triples completely disrupting the triple helix under our conditions: Cm•A-T, m^5^C•A-T, m^3^U•A-T, Am•A-T, m^6^A•A-T, I•A-T, and Gm•A-T (Table 1). It is perhaps not surprising that these modified noncanonical Z•A-T base triples completely destabilize the triple helix (Table 1; Fig. 1D), as our previous study showed that unmodified noncanonical Z•A-T base triples also had no observed triple helix formation (Kunkler et al. 2019). Additionally, the m^3^U•A-T base triple completely destabilizes our triple helix, likely due to the *N*^3^-methyl group disrupting Hoogsteen base pairing. Three U-modified Z•A-T base triples (m^5^U, Ψ, and s^4^U) destabilize the triple helix to ∼0.4 the stability of the unmodified U•A-T base triple (Table 1; Fig. 1D). Though we show a single m^5^U•A-T base triple destabilizes an R•D-D triple helix, a previous study used a UV thermal denaturation assay to show that multiple m^5^U•A-m^5^U base triples stabilized an R•R-R and an R•D-R triple helix, likely due to the increased base stacking ability of m^5^U base over uracil (Wang et al. 1997). To the best of our knowledge, Ψ and s^4^U have not been studied in the context of an R•D-D triple helix, though both poly(Ψ) and poly(s^4^U) can form R•R-R triple helices with poly(A) in the same 2:1 ratio as poly(U):poly(A) triple helices (Felsenfeld and Rich 1957; Felsenfeld et al. 1957; Simuth et al. 1970). It should be noted that even though s^4^U is not naturally occurring in humans, it is used in biochemical techniques to increase the UV-crosslinking efficiency without greatly altering the chemical properties of the RNA. However, the 2.8-fold decrease in our R•D-D triple helices indicates that the addition of s^4^U could lead to changes in R•D-D formation within these experiments. Herein, the Um•A-T base triple showed the highest stability with a *K*_D, EMSA_ of 132 ± 8 nM, minimally stabilizing the triple helix to ∼1.4—the stability of the unmodified U•A-T base triple (Table 1; Fig. 1D). Previous studies have also shown stabilizing effects for the 2′-*O*-methylation of the RNA third strand within an R•D-D triple helix via UV thermal denaturation studies and suggest the stabilization is due to the locking of the RNA into the 3'-*endo* conformation (Shimizu et al. 1992; Wang and Kool 1995). It should be noted that the previous studies had the third strand completely 2′-*O*-methylated (Shimizu et al. 1992; Wang and Kool 1995). Therefore, the minimal stabilizing effect measured herein might be greater with additional 2′-*O*-methyl nucleotides in the RNA third strand.

**TABLE 1.**
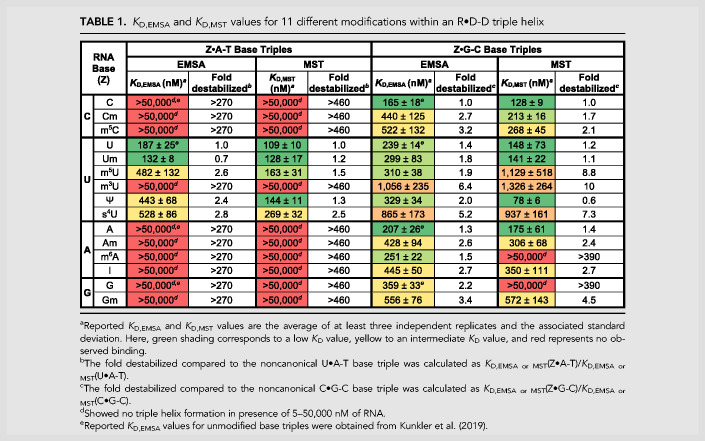
*K*_D, EMSA_ and *K*_D, MST_ values for 11 different modifications within an R•D-D triple helix

Using the same experimental setup, we determined *K*_D, EMSA_ values for the modified Z•G-C base triples (Table 1; Fig. 1E–H; Supplemental Table S1). All 11 modified Z•G-C base triples were able to form an R•D-D complex with *K*_D, EMSA_ values ranging from ∼250 to 1000 nM, showing that, unlike Z•A-T base triples, a single Z•G-C base triple can accommodate a wide variety of chemical moieties in the RNA strand (Table 1; Fig. 1D, H). Even the m^3^U•G-C base triple, which has a methyl group presumably interfering with the Hoogsteen interaction, had a *K*_D, EMSA_ value of 1056 ± 235 nM, only destabilizing the triple helix by 6.4-fold relative to the canonical C•G-C base triple and only 4.4-fold relative to the corresponding unmodified U•G-C base triple (*K*_D, EMSA_ = 239 ± 14 nM) (Table 1; Fig. 1H; Kunkler et al. 2019). Focusing on the modifications to the canonical C•G-C base triple, the Cm•G-C and m^5^C•G-C base triples had measured *K*_D, EMSA_ values of 440 ± 125 nM and 522 ± 132 nM, respectively, destabilizing the triple helix by 2.7- and 3.2-fold compared to the C•G-C base triple (Table 1; Fig. 1H). As mentioned before, the 2′-*O*-methyl modifications were previously shown to stabilize an R•D-D triple helix via UV thermal denaturation studies (Shimizu et al. 1992; Wang and Kool 1995). However, these other studies examined the stability of a triple helix composed of Um•A-T and Cm•G-C base triples in which the entire third strand is 2′-*O*-methylated rather than at a single position (Shimizu et al. 1992; Wang and Kool 1995). Therefore, it might be that the Cm•G-C base triple is stabilizing only in the presence of Um•A-T base triples, while a single Cm•G-C base triple is destabilizing, as shown herein (Table 1; Fig. 1H; Shimizu et al. 1992; Wang and Kool 1995). The m^5^C•G-C base triple has been used extensively to stabilize D•D-D triple helices (Lee et al. 1984; Povsic and Dervan 1989; Xodo et al. 1991). However, another study showed that the effect on the stability of triple helices with the m^5^C modification depends on the strand identity: stabilizing D•D-D triple helices while destabilizing R•R-R and R•D-R triple helices (Wang et al. 1997). To our knowledge, m^5^C•G-C base triples within an R•D-D triple helix have not been tested; our data indicate that R•D-D triple helices are destabilized by an m^5^C•G-C base triple (Table 1; Fig. 1H). It may be that m^5^C is stabilizing within a DNA third stand but destabilizing within an RNA third strand, though it is not clear if the reason is due to the neighboring thymine bases (which also contain a methyl group at position 5) or due to the differences in the ribose pucker. As for the modified noncanonical Z•G-C base triples, stabilities relative to C•G-C ranged from no significant change (Um•G-C, m^5^U•G-C, Ψ•G-C, and m^6^A•G-C), moderately destabilized between ∼2.5- to 3.5-fold (Am•G-C, I•G-C, and Gm•G-C), and destabilized greater than fivefold (m^3^U•G-C and s^4^U•G-C). To the best of our knowledge, there are no published reports examining the stability of RNA modifications within noncanonical R•D-D base triples. Like the previously studied noncanonical R•D-D base triples, unmodified Z•G-C base triples can tolerate a wide range of bases while Z•A-T base triples are more sensitive to nucleotide mismatches (Table 1; Fig. 1D, H; Kunkler et al. 2019).

Overall, our EMSA results show that the modified base triples either have no effect or destabilize an R•D-D triple helix relative to the canonical U•A-T and C•G-C base triples. It should be noted that the relative stability of the base triples likely changes under different buffer conditions and sequence contexts, including the length of the triple helix, the ratio of U•A-T to C•G-C base triples, and nearest-neighbor effects (Leitner and Weisz 2000; Leitner et al. 2000; Plum 2002). For example, it is known that C•G-C-containing triple helices are stabilized when the pH is lowered (e.g., pH 5) due to the protonation of C•G-C base triples to C^+^•G-C, leading to an additional hydrogen bond in each C^+^•G interaction (Felsenfeld and Rich 1957; Felsenfeld et al. 1957; Völker and Klump 1994; Plum and Breslauer 1995; Leitner et al. 1998, 2000; Plum 2002). For D•D-D triple helices, the p*K*_a_ of C•G-C base triples flanked by T•A-T base triples is higher than neighboring C•G-C base triples or terminal C•G-C base triples (Leitner et al. 2000). In fact, the measured p*K*_a_ value of C•G-C flanked by T•A-T base triples is 7.4, suggesting that the C•G-C base triples in our R•D-D construct are possibly protonated at pH 7 (Leitner et al. 2000). Therefore, it is still necessary to examine the formation of each predicted triple helix and the effect on triple helix formation in the presence and absence of an RNA modification. Regarding methodologies to measure *K*_D_ values, one major pitfall with using EMSAs to measure binding affinity is that, because EMSAs are a separation method, the experiment is no longer in equilibrium. Therefore, all measured *K*_D, EMSA_ values are apparent dissociation constants (*K*_D, app_) and not true dissociation constants (*K*_D_). To test if *K*_D, EMSA_ values accurately reflect true *K*_D_ values, we employed MST as a secondary method to measure *K*_D_ values.

### Measured binding affinities for our R•D-D triple helix are similar using EMSA and MST

Though the phenomenon of molecules moving across temperature gradients was described over 150 years ago, using this principle to measure binding affinities has been made possible via microscale thermophoresis (MST) (Ludwig 1856; Seidel et al. 2013). MST is unique from other binding methods because it does not require immobilization, it does not depend on large size changes between apo and complex states, it can be used with complex buffers, and it uses small sample volumes (Seidel et al. 2013). To perform MST, one binding partner is fluorescently labeled (herein, Cy5-labeled D-D) and mixed with the other unlabeled binding partner at varying concentrations (herein, RNA from ∼2–50,000 nM). After equilibrium is reached, samples are loaded into capillaries and inserted into an MST instrument. The fluorescence of the sample is monitored at the same location that an IR laser heats the sample. Due to the phenomenon of thermophoresis, the molecules move either toward or away from the laser, thus changing the local fluorescence over time, which is recorded by the MST instrument. Because molecules move differently through a temperature gradient depending on size, charge, hydration shell, and conformation, the fluorescently labeled binding partner moves differently between its bound and unbound states. Therefore, MST can measure true equilibrium dissociation constants (*K*_D, MST_) for a variety of interactions, including protein–protein, protein–nucleic acid, nucleic acid–nucleic acid, protein–small molecule, and nucleic acid–small molecule interactions, including monitoring the formation of an R•D-D pyrimidine-motif triple helix (Seidel et al. 2013; Maldonado et al. 2018). Using the same R•D-D construct (except 5′-end has Cy5-fluorophore rather than a [^32^P]-radiolabel) and buffer conditions used for EMSAs, we used MST to measure the relative stabilities of the same Z•A-T and Z•G-C base triples (where Z = C, Cm, m^5^C, U, Um, m^5^U, m^3^U, Ψ, s^4^U, A, Am, m^6^A, I, G, Gm) (Supplemental Fig. S1).

**FIGURE 2.**
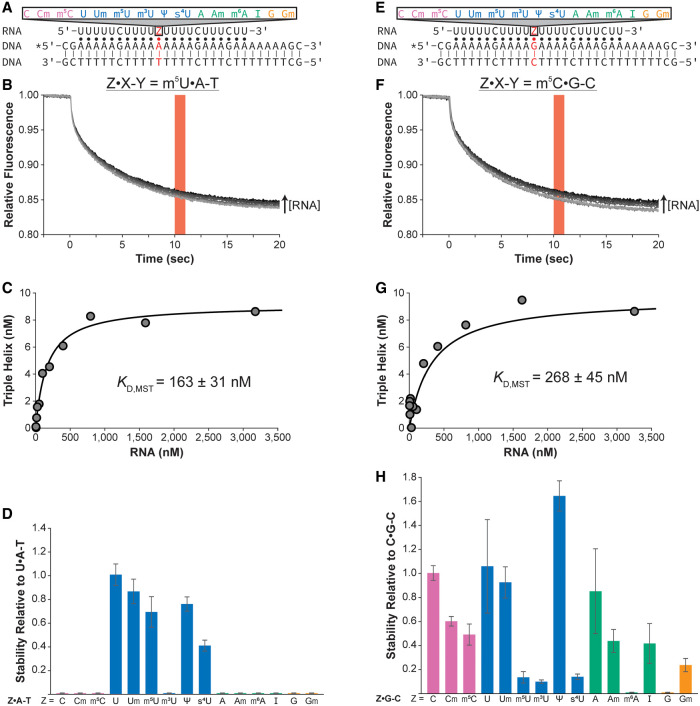
MST measurements for the relative stability of four unmodified and 11 modified RNA nucleotides in an R•D-D triple helix. Schematic depicting R•D-D triple helix with the varying position (*A*) Z•A-T and (*E*) Z•G-C in red. The putative Watson–Crick and Hoogsteen interactions are represented by a solid line (|) and a dot (•), respectively. The asterisk (*) denotes the location of Cy-5. Representative MST traces for the R•D-D triple helix containing (*B*) m^5^U•A-T base triple and (*F*) m^5^C•G-C base triple at Z•X-Y position. Traces go from light gray to black as the concentration of the RNA third strand is increased. Red bar shows the 10–11 sec time frame where all data are averaged. Representative binding curve generated using MST data points for R•D-D triple helix containing the (*C*) m^5^U•A-T and (*G*) m^5^C•G-C base triples. Please note that data points were collected up to at least 50,000 nM of the third strand RNA but are not shown in these plots for better visualization of the measurements near the *K*_D, MST_ (see Supplemental Fig. S2 for plot containing all data points). The relative stability of each (*D*) Z•A-T base triple and (*H*) Z•G-C base triple is shown as bar plots. The relative stability was calculated as (*D*) *K*_D, MST_(U•A-T)/*K*_D, MST_(Z•A-T) and (*H*) *K*_D, MST_(C•G-C)/*K*_D, MST_(Z•G-C). Each bar color represents a specific RNA nucleobase: pink for C, blue for U, green for A, and orange for G. Reported *K*_D, MST_ values and relative stability values are an average of at least three independent replicates and the associated standard deviation.

First, we examined the Z•A-T base triples using an R•D-D construct with a 5′-Cy5-labeled purine-rich DNA strand (Fig. 2A). A change in relative fluorescence was observed with increasing concentrations of the RNA strand, presumably binding to dsDNA to form an R•D-D triple helix (Fig. 2B). *K*_D, MST_ values were determined for each of the four unmodified and the 11 modified Z•A-T base triples (Table 1; Fig. 2C, D; Supplemental Fig. S2A; Supplemental Table S2). The *K*_D, EMSA and MST_ values measured for the U•A-T base triple are within twofold (187 ± 25 nM and 109 ± 10 nM, respectively), showing the measured *K*_D_ values agree for both methods (Table 1; Kunkler et al. 2019). Further, both EMSA and MST measured similar stabilities relative to the canonical U•A-T base triple (Table 1; Figs. 1D, 2D). Both methods showed that all unmodified noncanonical Z•A-T base triples, their corresponding modified Z•A-T base triples, and m^3^U•A-T all completely disrupted triple helix formation (Table 1; Figs. 1D, 2D; Kunkler et al. 2019). Further, both methods show that the Um•A-T base triple has no effect on stability and the s^4^U•A-T base triple destabilizes the triple helix to ∼0.4 the stability of the unmodified U•A-T base triple (Table 1; Figs. 1D, 2D). However, while EMSAs showed a mild destabilization of ∼2.5-fold for the m^5^U•A-T and Ψ•A-T base triples compared to the canonical U•A-T base triple, MST showed no significant difference in their relative stabilities (Table 1; Figs. 1D, 2D). Overall, the *K*_D, EMSA and MST_ values and the relative stability of each Z•A-T base triple are comparable for both methods.

We also tested the Z•G-C base triples using the same MST setup (Table 1; Fig. 2E–H; Supplemental Fig. S2B; Supplemental Table S2). Like the canonical U•A-T base triple, the *K*_D, EMSA and MST_ values for the canonical C•G-C base triple are within twofold: 165 ± 18 nM and 128 ± 9 nM, respectively (Table 1). Further, most of the modified Z•G-C base triples had similar destabilization relative to C•G-C in their corresponding method: Cm•G-C, m^5^C•G-C, U•G-C, Um•G-C, m^3^U•G-C, s^4^U•G-C, A•G-C, Am•G-C, I•G-C, and Gm•G-C (Table 1). Two base triples, m^5^U•G-C and Ψ•G-C, showed binding using both assays but had relative stabilities that differed by approximately fourfold, while two different base triples, m^6^A•G-C and G•G-C, which had *K*_D, EMSA_ values of 251 ± 22 nM and 359 ± 33 nM, respectively, had no observed binding using MST (Table 1; Fig. 2H). These differences in the *K*_D, EMSA and MST_ values are likely due to some of the challenges with using MST to monitor R•D-D triple helix formation, which are discussed below. Overall, with a few exceptions, measured *K*_D_ values are comparable between the two methods.

There are multiple advantages of using MST over EMSAs to measure binding affinities, such as MST measuring an in-solution *K*_D_ and rapid data collection (∼15 min/run). However, we encountered some challenges when using MST for our R•D-D triple helix experiments. First, the relative fluorescence (F_t_/F_0_) for both the unbound and the bound states is not predictable or consistent among base triples (see Fig. 2B, F). Therefore, two assumptions are made when analyzing the data: (1) a change in the relative fluorescence corresponds to the formation of a triple helix and not another structure (e.g., an RNA–DNA double helix via strand displacement) and (2) if the relative fluorescence reaches a plateau, then that plateau is assumed to be the fully bound complex. Additionally, there is not much change in the relative fluorescence of dsDNA to the R•D-D triple helix (∼0.01 units at the 10–11 sec time point), which reduces the signal-to-noise ratio (see Fig. 2B, F) and often leads to large errors in measured *K*_D, MST_ values among replicates (see Table 1). Finally, we noticed that the measured *K*_D, MST_ values were larger the longer the IR laser was on, suggesting the heating of the sample leads to the dissociation of the R•D-D complex to dsDNA and RNA. Therefore, though MST is a powerful technique to measure the *K*_D_ of an RNA–dsDNA complex, we suggest using another method, such as an EMSA or UV thermal denaturation assay, to monitor the formation of a triple helix.

Using either EMSA or MST, all RNA modifications could form the triple helix at the Z•G-C base triple site, though some of the Z•A-T base triples completely disrupt the R•D-D triple helix tested herein. In general, all tested modifications either have no significant effect on the stability or destabilize a triple helix. However, it is possible that one of the other naturally occurring RNA modifications not tested herein could significantly stabilize the formation of a triple helix. Further, RNAs with multiple modifications, modifications within a different sequence context, or modifications at a different location in the triple helix (i.e., not a central location) may have varying effects on the stability of an R•D-D triple helix. Overall, many of the RNA modifications examined destabilize the R•D-D triple helix, showing that minor changes to the RNA can perturb R•D-D triple helix formation.

### Known RNA modification sites in a human lncRNA destabilize an R•D-D triple helix in vitro

**FIGURE 3.**
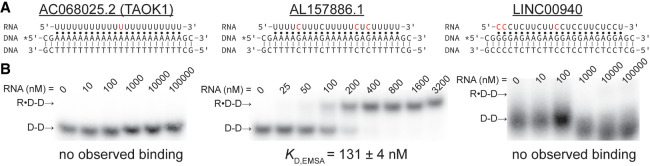
EMSA results for candidate lncRNA•gDNA triple helices. (*A*) R•D-D schematics show sequences and modification sites (red) of the 22-nt segments from the three lncRNAs examined: AC068025.2 (TAOK1), AL157886.1, and LINC00940. The putative Watson–Crick and Hoogsteen interactions are represented by a solid line (|) and a dot (•), respectively. The asterisk (*) denotes the location of the 5'-[^32^P]-radiolabel. (*B*) Representative gel images of dsDNA (D-D) and unmodified 22-nt segments from the three lncRNAs examined. Only the AL157886.1 segment (*center*) shows a shift from dsDNA (D-D) to triple helix (R•D-D) as increasing amounts of RNA are added. No binding to dsDNA was observed for the AC068025.2 (TAOK1) and LINC00940 segments (*left* and *right*, respectively). The *K*_D, EMSA_ value measured for AL157886.1•dsDNA is the average of four independent replicates and the associated standard deviation.

**FIGURE 4.**
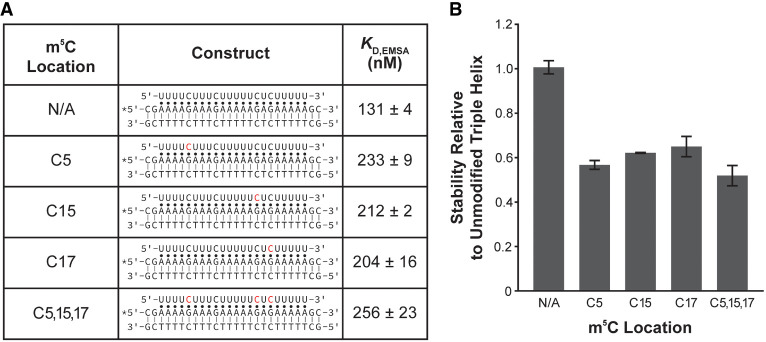
EMSA results for modified 22-nt sequence from lncRNA AL157886.1 binding to D-D. (*A*) Table showing the R•D-D constructs and measured *K*_D, EMSA_ values for the AL157886.1 segment containing no modifications, a single m^5^C modification at three different sites, and three m^5^C modifications. Red nucleotides indicate the m^5^C location in each RNA. The putative Watson–Crick and Hoogsteen interactions are represented by a solid line (|) and a dot (•), respectively. The asterisk (*) denotes the location of the 5′-[^32^P]-radiolabel. The reported *K*_D, EMSA_ values are average of at least three independent replicates and the associated standard deviation. (*B*) Bar plot showing the stability of each modified triple helix relative to the unmodified triple helix. The relative stability was calculated as *K*_D, EMSA_(unmodified)/*K*_D, EMSA_(modified). Error bars are the standard deviation of at least three independent experiments.

RNA modifications affect the stability of R•D-D triple helices based on the results of our model R•D-D construct presented herein. Therefore, we searched for potential biologically relevant examples of an R•D-D triple helix being regulated by a modification switch. First, using RMVar, we examined published, high-confidence transcriptome-mapped modification sites (m^5^C, m^5^U, Ψ, I, and Nm) within a pyrimidine-rich segment of human lncRNAs that are at least 19-nt long, as our previous study showed a minimum of 19 base triples are required for triple helix formation in vitro (Supplemental File S1; Kunkler et al. 2019; Luo et al. 2021). Please note that m^6^A was not included in our search as high-confidence sites are within the DRACH-motif (where D = A/G/U, R = A/G, H = A/C/U) and are therefore not within pyrimidine-rich sequences (Wei and Moss 1977; Narayan and Rottman 1988; Csepany et al. 1990; Harper et al. 1990; Dominissini et al. 2012; Meyer et al. 2012). Three lncRNAs fulfilled the aforementioned criteria: AC068025.2 (TAOK1), AL157886.1, and LINC00940 (Fig. 3A). Further, Triplexator predicts that the RNA segment of both AL157886.1 and LINC00940 may form triple helices within promotor regions (Supplemental File S2; Buske et al. 2012). Therefore, we performed EMSAs to monitor the formation of the triple helix using only the region-of-interest, for each lncRNA and DNA contain only canonical U•A-T and C•G-C base triples (Fig. 3; Supplemental Table S3). Of the three lncRNA segments tested, only the AL157886.1 segment showed triple helix formation (*K*_D, EMSA_ = 131 ± 4 nM), demonstrating the importance of experimentally testing predicted triple helices (Fig. 3). The lack of triple helix formation for the AC068025.2 and LINC00940 segments is perhaps not too surprising, as previous studies show that pyrimidine-motif triple helices are unstable without C•G-C base triples (as in AC068025.2), with a high ratio of C•G-C to U•A-T (as in LINC00940), and when neighboring base triples are C•G-C base triples (as in LINC00940) (Roberts and Crothers 1996; James et al. 2003; Maldonado et al. 2018). Because the unmodified AL157886.1 segment showed triple helix formation, we examined the stability of the AL157886.1•dsDNA triple helix with one m^5^C modification at each of the three biologically relevant locations (C5, C15, and C17) and with m^5^C at all three of the biologically relevant locations (Fig. 4). Compared to the unmodified AL157886.1 segment, the three single m^5^C-modified AL157886.1 segments showed an average relative destabilization to only ∼0.6, while the triple m^5^C-modified AL157886.1 segment destabilized to ∼0.5 (Fig. 4). Though the AL157886.1•dsDNA triple helix was only modestly destabilized by the presence of m^5^C modifications, this example gives precedence to the possibility of lncRNA•gDNA triple helix formation being regulated by RNA modifications in vivo. However, further studies are needed to confirm if the formation of AL157886.1•gDNA or other R•D-D triple helices are regulated by an RNA modification in cells.

Beyond the observed formation of an R•D-D triple helix in vitro, there are some important considerations before suggesting a modification can alter the stability of a cellular R•D-D triple helix. First, for any cellular R•D-D triple helix, it is essential that the RNA of interest is localized to the nucleus where it can interact with gDNA and that the binding sites in both the lncRNA and the gDNA are accessible. Unfortunately, the localization of most lncRNAs, including AL157886.1, is unknown (Mas-Ponte et al. 2017; Wen et al. 2018). For modified cellular R•D-D triple helices containing nuclear-localized RNAs, the enzyme responsible for the modification must also localize within the nucleus. For the 11 RNA modifications tested herein, the enzymes responsible for the m^5^C, Ψ, m^6^A, and I modifications in lncRNAs are present within the nucleus, but the enzymes responsible for m^5^U and Nm in lncRNAs and their subcellular localization are not known (Savva et al. 2012; Liu et al. 2013; Ping et al. 2014; Aguilo et al. 2016; Zhao et al. 2018; Bohnsack et al. 2019). Other points of consideration for modified lncRNAs are the nuclear abundance and if there are differences in the ratio of modified to unmodified nucleotide at the site of interest within different cell types, disease states, or developmental states, though a recent study suggests that lncRNAs can overcome the limitation of low-expression through the formation of phase-separated bodies (Unfried and Ulitsky 2022). Also notable is that we examined R•D-D triple helices with a 19-nt cut-off due to their formation in vitro, although shorter triple helices may occur in vivo due to the cellular microenvironment, such as the presence of nucleosomes (Kunkler et al. 2019; Maldonado et al. 2019; Unfried and Ulitsky 2022). Beyond the other lncRNA modifications not tested herein (e.g., 1-methyladenosine and *N*^6^,2′-*O*-dimethyladenosine) that may lead to changes in the stability of R•D-D triple helices in vivo, DNA modifications may also alter the stability of R•D-D triple helices (Sood et al. 2019). 5-methylcytosine (5mC), the most prevalent DNA modification in humans, is likely to affect the stability of R•D-D triple helices in vitro. Though 5mC would not disrupt hydrogen-bonding interactions with the RNA third strand, 5mC has been shown to change the stability and structure of dsDNA in vitro, such as increased thermal stability, increased base-flipping, and B-to-Z-form double helix transformations, which could affect R•D-D triple helix formation (Yang et al. 2020a; Furukawa et al. 2021). However, 5mC is present in vivo in promoter regions (i.e., CpG islands) in human gDNA within the CG-motif, not in pyrimidine-rich sequences with high triple helix-forming potential (Jones 2012). Therefore, though 5mC likely affects the formation of R•D-D triple helices in vitro, it is unlikely that 5mC marks are in triple helix-forming regions in vivo. Other DNA modifications, such as 7-methyladenosine and 7-methylguanosine, which both contain a methyl group on the Hoogsteen face of the purine, would likely destabilize R•D-D triple helices. Another way a nucleotide modification could alter the formation of R•D-D triple helices in vivo is by altering the accessibility of the triple helix-forming site directly through the recruitment of protein modification “readers” that directly block the site, or indirectly through chromatin remodeling and changes in intramolecular RNA secondary structures. For example, UHRF1 (ubiquitin-like with PHD and ring finger domains 1) interacts with 5mC in dsDNA, which would make the DNA inaccessible at that location by precluding the formation of an R•D-D triple helix (Arita et al. 2008). In addition to enzyme-mediated nucleotide modifications, some modifications on both DNA and RNA occur through nonenzymatic reactions. For instance, oxidative stress, toxins, UV damage, and chemotherapeutics can lead to spontaneous modifications, which could also alter the formation of R•D-D triple helices (de Bont and van Larebeke 2004). Overall, both enzymatic and nonenzymatic nucleic acid modifications may influence R•D-D triple helix formation in cells.

In summary, there are multiple considerations to be made before concluding a lncRNA•gDNA triple helix is forming in vivo and a modification in the RNA (or DNA) might alter the formation of the lncRNA•gDNA triple helix. Herein, we show that modified R•D-D base triples at a single position within an R•D-D triple helix can destabilize an R•D-D triple helix in vitro, from minor destabilization to completely disrupting the R•D-D triple helix formation. However, whether RNA modifications regulate the formation of cellular R•D-D triple helices remains to be explored using cell-based assays.

## MATERIALS AND METHODS

### Oligonucleotide preparation

Oligonucleotides were chemically synthesized and purchased as follows: DNA and unmodified RNA from Sigma-Aldrich and modified RNA from Dharmacon/Horizon Discovery. Oligonucleotide sequences are shown in Figures 1A, E, 2A, E, 3, and 4A. For electrophoretic mobility shift assays, oligonucleotides were prepared as before (Kunkler et al. 2019). Briefly, the purine-rich DNA oligonucleotides were 5′-end radiolabeled using γ-[^32^P]-ATP (Perkin Elmer) and T4 PNK (New England Biolabs) per the manufacturer's protocol. Unreacted γ-[^32^P]-ATP was removed using a G25 MicroSpin Column (GE Healthcare). For microscale thermophoresis, the chemically synthesized 5′-Cy5-labeled purine-rich DNA was purchased from Sigma-Aldrich.

### Electrophoretic mobility shift assays (EMSAs)

The procedure was performed as in our prior study of R•D-D triple helix stability (Kunkler et al. 2019). Briefly, 10 nM of a pyrimidine-rich 31-mer DNA oligonucleotide and a 10 nM of the complementary purine-rich 5′-[^32^P]-radiolabeled 31-mer DNA oligonucleotide were mixed in Binding Buffer (25 mM sodium cacodylate [pH 7], 125 mM NaCl, 2 mM MgCl_2_, 10% glycerol, and 0.1 mg/mL yeast tRNA) before heating at 95°C for 2 min and snap-cooling on ice for 2 min to form dsDNA. The pyrimidine-rich 22-mer RNA oligonucleotide was added at increasing amounts (10–100,000 nM) and equilibrated at 4°C for 24 h. Samples were loaded onto a 12% native polyacrylamide gel (19:1 acrylamide:bisacrylamide, 40 mM Tris-acetate at pH 7.0, 1 mM EDTA, and 10 mM MgCl_2_) and resolved at 195 V for ∼6 h at 4°C. Gels were then wrapped in plastic wrap and exposed overnight to a phosphorimager screen. The following day, the screens were scanned using an Amersham Typhoon (GE Healthcare) and quantified using ImageQuant software (GE Healthcare). A plot of triple helix concentration versus the RNA concentration was fit to the Hill equation (Equation 1) using OriginPro 2021 Graphing Software (OriginLab Corporation):

(1) $$[\mathrm{ts}]=[\mathrm{ds}]{\left[\mathrm{ss}\right]}^{n}/(K_{D,\mathrm{EMSA}}^{\;\;\;n}+{\left[\mathrm{ss}\right]}^{n}).$$

In Equation 1, [ts] is the triple helix concentration, [ds] is the initial Watson–Crick dsDNA concentration, [ss] is the initial RNA concentration, *K*_D, EMSA_ is the apparent equilibrium dissociation constant, and *n* is the degree of cooperativity. Here, all parameters ([ds], *K*_D, EMSA_, *n*) were treated as variables. Extrapolated values for each base triple are in Table 1, Supplemental Tables S1 and S3, and Figure 4A.

### Microscale thermophoresis (MST)

In Binding Buffer, 10 nM of a pyrimidine-rich 31-mer DNA oligonucleotide was mixed with 10 nM of its complementary purine-rich 5′-Cy5-labeled 31-mer DNA oligonucleotide. T­he solution was heated at 95°C for 2 min before snap-cooling on ice to form dsDNA. The pyrimidine-rich 22-mer RNA oligonucleotide was added at decreasing twofold serial dilutions from 51.2 µM to ∼2 nM. After a 24-h equilibration at 4°C, the samples were analyzed on the NanoTemper Monolith nt. 155 Pico (NanoTemper Tech) at room temperature using Monolith standard capillaries at 5% excitation power and low MST power. Data were collected using MO.Control v.1.6.1 and MO.Affinity Analysis v2.3 (NanoTemper Tech). We noticed that measured *K*_D, MST_ values were larger the longer the IR laser was powered on, likely due to temperature increase inducing dissociation of the R•D-D complex. Therefore, rather than allowing the MO.Affinity Analysis software to choose the time with the best signal-to-noise ratio, all experiments averaged the relative fluorescence at the 10–11 sec time point (F_10–11_/F_0_). Averaged relative fluorescence for each run was normalized from 0 to 10 nM triple helix. A plot of triple helix concentration versus the RNA concentration was fit to the quadratic equation (Equation 2) using OriginPro 2021 Graphing Software (OriginLab Corporation):

(2) $$[\text{ts}]=0.5(K_{D,\mathrm{MST}}+[\text{ds}]+[\text{ss}])-0.5{\left({\left(K_{D,\mathrm{MST}}+[\text{ds}]+[\text{ss}]\right)}^{2}-4[\mathrm{ds}][\mathrm{ss}]\right)}^{0.5}.$$

In Equation 2, [ts] refers to the initial triple helix concentration, [ds] is the initial Watson–Crick dsDNA concentration, [ss] is the initial RNA concentration, and *K*_D, MST_ is the equilibrium dissociation constant. Here, all parameters ([ds], *K*_D, MST_) were treated as variables. Extrapolated values for each base triple are in Table 1 and Supplemental Table S2.

### In silico prediction of modified R•D-D triple helices

A list of human lncRNAs containing high-confidence modifications that we examined herein (m^5^C, m^5^U, Ψ, I, and Nm), and the location of the modification within the lncRNA (see Supplemental File S1) was compiled on February 1, 2022 using RMVar (https://rmvar.renlab.org) (Luo et al. 2021). Please note that m^3^U and s^4^U have not been detected in human lncRNAs and that m^6^A was not examined because the m^6^A mark in human lncRNAs occurs primarily in the DRACH-motif, which has multiple purines that would destabilize pyrimidine-motif R•D-D triple helices (Wei and Moss 1977; Narayan and Rottman 1988; Csepany et al. 1990; Harper et al. 1990; Dominissini et al. 2012; Meyer et al. 2012; Kunkler et al. 2019). Next, RNA sequences surrounding the modification site were examined for pyrimidine-rich sequences with a high potential to form R•D-D triple helices. RNAs that had a modification within a pyrimidine-rich sequence at least 19-nt long, the previously reported shortest length of a stable pyrimidine motif R•D-D triple helix under our binding conditions, were tested for triple helix formation in their unmodified states using the same EMSA conditions as above (Kunkler et al. 2019). Further, Triplexator was used to predict if the RNA segments have the potential to bind within human promotor regions, as performed previously (Buske et al. 2012; Kunkler et al. 2019). Briefly, the RNA segments (see Fig. 3A) and all human promotor sequences (GRCh38, version Eldorado 04-2021; accessed March 7, 2022; www.genomatix.de) were inserted as the single- and double-stranded sequence, respectively, using Triplexator on its default settings for all parameters (Buske et al. 2012) (see Supplemental File S2). Please note that to compile Triplexator v.1.3.2 from source code successfully, the libboost_iostreams-mt.so file integrated with Linux 3.10.0 or above was symlinked to an empty file named libboost_iostreams-mt.so.5.

## SUPPLEMENTAL MATERIAL

Supplemental material is available for this article.

## Supplementary Material

Supplemental Material

## Figures and Tables

**Charlotte Kunkler:**
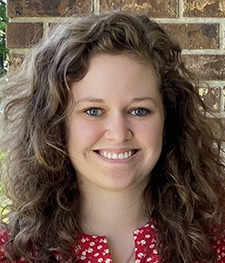


## MEET THE FIRST AUTHOR

***Meet the First Author(s)* is a new editorial feature within *RNA*, in which the first author(s) of research-based papers in each issue have the opportunity to introduce themselves and their work to readers of *RNA* and the RNA research community. Charlotte Kunkler is the first author of this paper, “A single natural RNA modification can destabilize a U•A-T-rich RNA•DNA-DNA triple helix.” Charlotte is a biochemistry graduate student in the Jessica A. Brown laboratory at the University of Notre Dame.**

**What are the major results described in your paper and how do they impact this branch of the field?**

RNA modifications have previously been shown to alter the stability of RNA structures. Herein, we examined the stability of 11 different naturally occurring RNA modifications at a single location within a U•A-T-rich pyrimidine-motif RNA•DNA-DNA triple helix. Our results indicate that, for the modifications tested, RNA modifications either destabilize or have no significant effect on the stability of the triple helix. Therefore, it is important to consider the modification status of an RNA predicted to form an RNA•DNA-DNA triple helix, as modifications can affect RNA•DNA-DNA triple helix stability.

**What led you to study RNA or this aspect of RNA science?**

The structure/function relationship of RNAs fascinates me. As a graduate student, I decided to study one particular RNA structure, the triple helix, to better understand the base triples that stabilize the triple-helical structure and how altering the formation of triple helices might lead to a change in function.

**During the course of these experiments, were there any surprising results or particular difficulties that altered your thinking and subsequent focus?**

I was surprised to find three different human biological examples that contain a modification within a pyrimidine-rich sequence. The original idea of searching for these sequences was a “shot in the dark, ” but I am pleased that the search yielded fruitful results. I am excited to see if the examples we found or other RNA modifications within RNA•DNA-DNA triple helices have a functional change in cells.

**What are some of the landmark moments that provoked your interest in science or your development as a scientist?**

Growing up, I viewed science as any other school subject where a student learns facts about the subject from text written by experts. However, during my undergrad studies, I joined a research group where I was first introduced to using science as a tool for discovery. Science fascinates me because it is dynamic, with endless opportunity to learn and to contribute to the pool of knowledge. Giving back to my community is important to me, and science allows me to share what I have learned at the bench through the publication of my findings.

**If you were able to give one piece of advice to your younger self, what would that be?**

It is okay to be uncomfortable in science. Failed experiments, unexpected results, and seemingly contradictory conclusions are not a reflection on your worth as a person or scientist. Rather, uncomfortable situations are nucleation sites for personal growth and scientific discoveries with even greater impact.
