# Supplementary material for: A single natural RNA modification can destabilize a U•A-T-rich RNA•DNA-DNA triple helix
Source: RNA. 2022 Sep;28(9):1172–84. doi: 10.1261/rna.079244.122 (PMC9380742; doi:10.1261/rna.079244.122)
Supplement: Supplemental Material [file supp_28_9_1172__DC1.html]

Supplemental Material 

# A single natural RNA modification can destabilize a U•A-T-rich RNA•DNA-DNA triple helix

## Supplemental Material

- Supplemental\_Material.pdf
- Supplemental\_File\_S1\_lncRNA\_candidates.xlsx
- Supplemental\_File\_S2\_Triplexator\_Results.xlsx
