# Supplementary material for: A single natural RNA modification can destabilize a U•A-T-rich RNA•DNA-DNA triple helix
Source: RNA. 2022 Sep;28(9):1172–84. doi: 10.1261/rna.079244.122 (PMC9380742; doi:10.1261/rna.079244.122)
Supplement: Supplemental Material [file supp_079244.122_Supplemental_Material.pdf]

SUPPLEMENTAL MATERIAL

A single RNA modification destabilizes an RNA•DNA-DNA triple helix

Charlotte N. Kunkler, Grace E. Schiefelbein, Nathan J. O'Leary, Phillip J. McCown, Jessica A. Brown

Supplemental Tables

Supplemental Table S1: Extrapolated parameters for R•D-D triple helices via EMSAs.

RNA Base (Z)[a]		Z•A-T Base Triples[a]		Z•G-C Base Triples[a]	
		Concentration of dsDNA (nM)[b]	Degree of Cooperativity[c]	Concentration of dsDNA (nM)[b]	Degree of Cooperativity[c]
**C**	C	n/a[d,e]	n/a[d,e]	8.9 ± 1.0[e]	3.6 ± 0.5[e]
	Cm	n/a[d]	n/a[d]	9.1 ± 0.3	2.7 ± 0.4
	m^{5}C	n/a[d]	n/a[d]	9.2 ± 0.3	2.8 ± 0.3
**U**	U	9.4 ± 0.9[e]	2.2 ± 0.1[e]	7.5 ± 1.6[e]	2.0 ± 0.4[e]
	Um	8.1 ± 0.3	1.9 ± 0.1	7.8 ± 0.2	2.0 ± 0.2
	m^{5}U	8.3 ± 0.2	1.5 ± 0.2	6.6 ± 0.4	1.7 ± 0.1
	m^{3}U	n/a[d]	n/a[d]	9.5 ± 0.1	1.9 ± 0.2
	Ψ	8.0 ± 0.3	1.9 ± 0.4	9.7 ± 0.1	2.2 ± 0.2
	s^{4}U	7.2 ± 1.4	1.9 ± 0.5	8.9 ± 0.2	1.3 ± 0.2
**A**	A	n/a[d,e]	n/a[d,e]	8.8 ± 0.1[e]	2.2 ± 0.1[e]
	Am	n/a[d]	n/a[d]	8.3 ± 0.3	1.9 ± 0.1
	m^{6}A	n/a[d]	n/a[d]	6.9 ± 0.2	1.7 ± 0.1
	I	n/a[d]	n/a[d]	8.8 ± 0.1	2.1 ± 0.1
**G**	G	n/a[d,e]	n/a[d,e]	8.7 ± 0.4[e]	2.2 ± 0.3[e]
	Gm	n/a[d]	n/a[d]	7.0 ± 0.2	2.5 ± 0.4

[a] Base triples follow Z•X-Y notation, where Z is the RNA third strand base and X-Y is the Watson-Crick dsDNA base pair. Schematics of R•D-D triple helix constructs can be found in **Figures 1A** and **1E**, respectively.
[b] Measured concentration of dsDNA ("[ds]" in **Equation 1**) based on amount bound to the third strand forming a triple helix. Reported values are the average of at least three independent experiments ± standard deviation.
[c] Measured degree of cooperativity ("*n*" in **Equation 1**) reported as the average of at least three independent experiments ± standard deviation.
[d] Not applicable because no binding of the RNA third strand to dsDNA was observed.
[e] Reported values for unmodified base triples were obtained from reference (1).

Supplemental Table S2: Extrapolated parameters for R•D-D triple helices via MST.

RNA Base (Z)[a]		Z•A-T Base Triples[a]	Z•G-C Base Triples[a]
		Concentration of dsDNA (nM)[b]	Concentration of dsDNA (nM)[b]
C	C	n/a[c]	9.8 ± 0.1
	Cm	n/a[c]	9.7 ± 0.4
	m^5C	n/a[c]	9.7 ± 0.3
U	U	9.9 ± 0.7	8.3 ± 0.2
	Um	8.6 ± 0.1	8.2 ± 0.4
	m^5U	9.3 ± 0.3	8.6 ± 0.5
	m^3U	n/a[c]	10.0 ± 0.2
	Ψ	9.5 ± 0.2	8.7 ± 0.3
	s^4U	8.1 ± 0.4	9.5 ± 0.5
A	A	n/a[c]	9.0 ± 0.2
	Am	n/a[c]	8.2 ± 0.6
	m^6A	n/a[c]	n/a[c]
	I	n/a[c]	7.4 ± 0.3
G	G	n/a[c]	n/a[c]
	Gm	n/a[c]	9.0 ± 0.1

[a] Base triples follow Z•X-Y notation, where Z is the RNA third strand base and X-Y is the Watson-Crick dsDNA base pair. Schematics of R•D-D triple helix constructs are presented in **Figures 1A** and **1E**, respectively.

[b] Measured concentration of dsDNA ("[ds]" in **Equation 2**) based on amount bound to the third strand forming a triple helix. Reported values are the average of at least three independent experiments ± standard deviation.

[c] Not applicable because no binding of the RNA third strand to dsDNA was observed.

Supplemental Table S3: Extrapolated parameters for potential lncRNA•gDNA modification switches measured via EMSAs.

lncRNA segment[a]	Location of Modification [b]	Concentration of dsDNA (nM)[c]	Degree of Cooperativity[d]
AC068025.2 (TAOK1)	unmodified	n/a[e]	n/a[e]
AL157886.1	unmodified	9.8 ± 0.1	2.1 ± 0.1
	C5	9.9 ± 0.2	2.0 ± 0.1
	C15	9.7 ± 0.1	2.0 ± 0.1
	C17	9.6 ± 0.1	2.0 ± 0.1
	C5,15,17	10.0 ± 0.1	2.0 ± 0.1
LINC00940	unmodified	n/a[e]	n/a[e]

[a] lncRNA which contains the 22-nt pyrimidine-rich region of interest. R•D-D constructs are shown in **Figures 3A** and **4A**.
[b] Location of modification within the 22-nt lncRNA segment.
[c] Measured concentration of dsDNA ("[ds]" in **Equation 1**) based on amount bound to the third strand forming a triple helix. Reported values are the average of at least three independent experiments ± standard deviation.
[d] Measured degree of cooperativity ("*n*" in **Equation 1**) reported as the average of at least three independent experiments ± standard deviation.
[e] Not applicable because no binding of the RNA third strand to dsDNA was observed.

Supplemental Figures

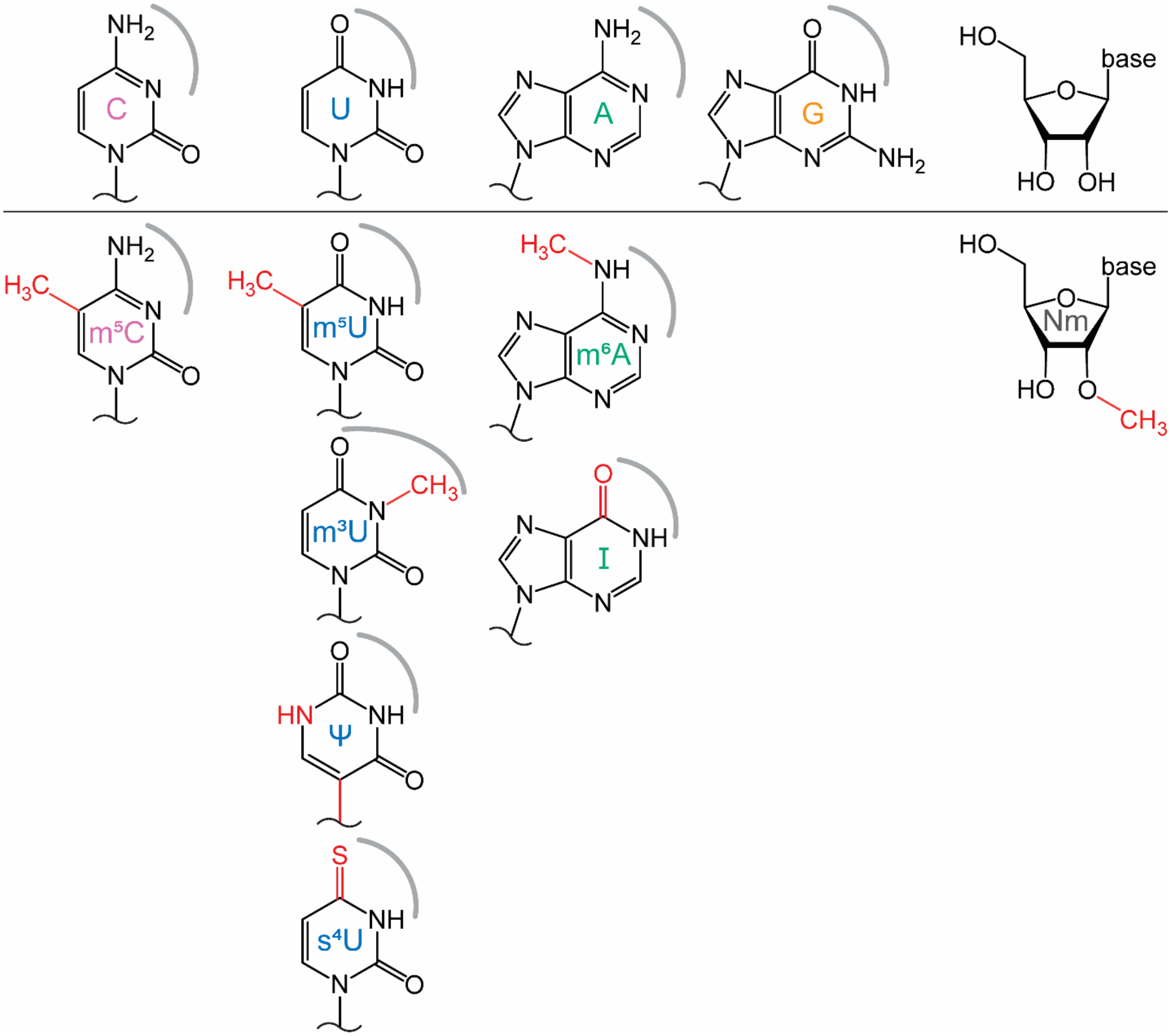

Supplemental Figure S1. Structures of unmodified and modified RNA nucleosides. Structures of each of the four unmodified RNA nucleosides (top row) and the eleven modified RNA nucleosides that were examined in this study. Each color represents a specific RNA nucleobase and its associated modified nucleoside: pink for C, blue for U, green for A, and orange for G. Red moieties denote the chemical modification. Gray arcs indicate the face where the RNA base has the potential to interact with the purine-rich DNA strand.

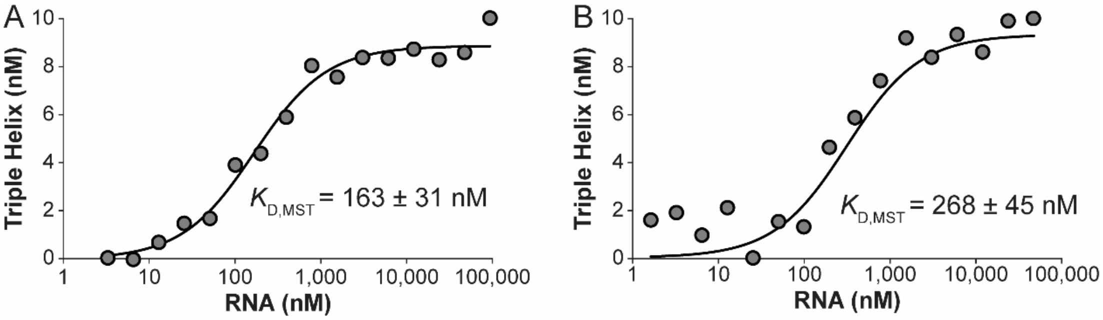

Supplemental Figure S2. Representative MST binding curves. Plot of all sixteen data points at time 10-11 sec for a single MST experiment when the varying base triple is A) m^5U•A-T and B) m^5C•G-C. Reported $K_{D,MST}$ values are an average of at least three independent replicates and the associated standard deviation.

Supplemental Files

Supplemental File S1. Human lncRNAs that contain high-confidence modification sites near pyrimidine-rich regions. Using the high-confidence modification sites downloaded from RMVar (https://rmvar.renlab.org) (2), we manually curated modification sites located within or near pyrimidine-rich regions. This file contains multiple sheets. “Best Candidates” includes the overall best candidates for potential modification-regulated R•D-D triple helices with bolded entries indicating candidates that were experimentally examined herein. “Nm”, “pseudoU", “m5C", “m5U", and “inosine” sheets contain candidate lncRNAs for each of the indicated modifications, with bolded entries indicating the best candidates. All sheets contain the same column headers derived directly from RMVar: “Modification Type”, “Chromosome Position” (of the modification), “Source” (i.e. method for modifications detection), “Species”, “Gene”, “Genetic Variants” (in different reference genomes), “SNP Position” (of the genetic variant), “Database” (that data were originally deposited into), “Transcriptome Regulation”, “Disease”, “Nucleotide Ref Seq” and “Nucleotide Alt Seq” (with SNP). Red and blue nucleotides in the “Nucleotide Ref Seq” and “Nucleotide Alt Seq” columns indicate the modification site and gene variation site(s), respectively. Please note that there were no predicted lncRNAs containing potential inosine switches.

Supplemental File S2. Triplexator results listing putative sites for lncRNA segments binding to human promoter DNA. Triplexator output files for each of the three 22-nt lncRNA segments to human promotor regions, separated by lncRNA of interest: “AC068025.2 (TAOK1)”, “AL157886.1”, and “LINC00940” (3). All sheets contain the same column headers derived directly from Triplexator: “TFO start” (start position of the RNA segment), “TFO end” (end position of the RNA segment”, “Duplex-ID” (promotor gene name derived from Genomatix), “TTS start” (start position of promotor DNA), “TTS end” (end position of promotor DNA), “Score” (number of canonical U•A-T/C•G-C bases triples), “Error-rate” (% noncanonical base triples), “Errors” (type and location of noncanonical base triples), “Motif” (all “Y” for pyrimidine-motif), “Strand” (strand of promotor DNA providing the purine-rich tract), “Orientation” (all “P” for parallel), and “Guanine-rate” (ratio of C•G-C to all canonical base triples). Only predicted triple helices of at least 19-nts in length with an error of no more than one non-canonical base triple (<0.05) are included. Please note that there were no predicted sites for AC068025.2 (TAOK1).

References

1. Kunkler,C.N., Hulewicz,J.P., Hickman,S.C., Wang,M.C., McCown,P.J. and Brown,J.A. (2019) Stability of an RNA•DNA–DNA triple helix depends on base triplet composition and length of the RNA third strand. *Nucleic Acids Research*, **47**, 7213–7222.
2. Luo,X., Li,H., Liang,J., Zhao,Q., Xie,Y., Ren,J. and Zuo,Z. (2021) RMVar: An updated database of functional variants involved in RNA modifications. *Nucleic Acids Research*, **49**, D1405–D1412.
3. Buske,F.A., Bauer,D.C., Mattick,J.S. and Bailey,T.L. (2012) Triplexator: Detecting nucleic acid triple helices in genomic and transcriptomic data. *Genome Res*, **22**, 1372–81.
